# Taking what you get or Getting what you Need: A Qualitative Study on Experiences with Mental Health and Welfare Services in Long-Term Recovery in First-Episode Psychosis

**DOI:** 10.1007/s10597-024-01356-6

**Published:** 2024-10-16

**Authors:** Gina Åsbø, Hanne Haavind, Sindre Hembre Kruse, Kristin Fjelnseth Wold, Wenche ten Velden Hegelstad, Kristin Lie Romm, Mike Slade, Torill Ueland, Ingrid Melle, Carmen Simonsen

**Affiliations:** 1 Section for Clinical Psychosis Research, Department of Research and Innovation, Division of Mental Health and Addiction, Oslo University Hospital, Oslo, Norway; 2 Early Intervention in Psychosis Advisory Unit for Southeast Norway, Division of Mental Health and Addiction, Oslo University Hospital, Oslo, Norway; 3 Department of Psychology, Faculty of Social Sciences, University of Oslo, Oslo, Norway; 4 Institute of Clinical Medicine, University of Oslo, Oslo, Norway; 5 TIPS – Centre for Clinical Research in Psychosis, Stavanger University Hospital, Stavanger, Norway; 6 Faculty of Social Sciences, University of Stavanger, Stavanger, Norway; 7 School of Health Sciences, Institute of Mental Health, University of Nottingham, Nottingham, UK; 8 Faculty of Nursing and Health Sciences, Health and Community Participation Division, Nord University, Namsos, Norway

**Keywords:** First-episode psychosis, Schizophrenia, Bipolar disorder, Services, Recovery, Qualitative

## Abstract

How people in long-term recovery (clinical and personal) in first-episode psychosis (schizophrenia and bipolar spectrum disorders) experience the mental health and welfare services they interact with is not frequently studied but has significant implications. We therefore aimed to explore which aspects of these services people with FEP evaluate as important for their long-term recovery. Twenty participants in clinical and/or personal recovery from two Norwegian long-term follow-up studies after FEP (TOP 10-year and TIPS 20-year) were sampled for this interview-based qualitative study. The research-team included service user experience. A deductive analysis based on personal accounts of recovery generated five service aspects. Few specific types of interventions were reported to promote recovery although medications, psychotherapy and employment support were mentioned. Participants valued services based in collaboration and that focused on their resources rather than limitations. The importance of long-term follow-up with a consistent aim was highlighted, as was the inclusion of caregivers and peers. Welfare services contributed to recovery by supporting basic needs and safety, but some experienced social exclusion when not participating in the labor market. This study is unique in exploring the role of services, including welfare, in long-term FEP recovery from service user perspectives. Participants evaluated that services played a more indirect role in long-term recovery by supporting their personal resources, although what they needed from services had frequently not been offered. Their expertise by experience contributes valuable knowledge. Better service coordination and consistent implementation of this knowledge are crucial to support recovery in FEP.

**Supplementary Information:**

The online version contains supplementary material available at 10.1007/s10597-024-01356-6.

## Introduction

Promoting long-term recovery in first-episode psychosis (FEP) is both a goal and a challenge for services (Britz & Jones, 2023; Hansen et al., 2022). There is growing evidence regarding facilitators of recovery that could aid services, but there are some limitations to this research.

First, studies on recovery have mainly been concerned with improving *clinical recovery*, defined as symptom remission and adequate functioning (Andreasen et al., 2005; Hansen et al., 2022). This is a contrast to what service users view as equally or more important to them, namely *personal recovery*, defined as experienced connectedness, hope, identity, meaning and empowerment (CHIME) (Leamy et al., 2011; Skar-Fröding et al., 2021; Slade, 2009).

Furthermore, recovery and treatment in psychotic disorders have primarily been investigated *quantitatively*. *Qualitative* research on personal recovery has largely been focused on individual-related facilitators, such as agency (Boydell et al., 2010; Leendertse et al., 2021; Temesgen et al., 2019; Wood & Alsawy, 2018). Studies on treatment-related faciliators are most often concerned with whether types of treatment, such as medication or psychoeducation, positively or negatively affect personal recovery (Temesgen et al., 2019; Wood & Alsawy, 2018). Nonetheless, people with FEP typically receive extensive treatment and interact with many services which are often a significant part of their social context and recovery process (Fusar-Poli et al., 2022; Topor et al., 2011). More research on the role of services in FEP recovery from the perspective of service users is therefore needed (Fusar-Poli et al., 2022).

Additionally, research on the relationship between services and recovery is almost exclusively focused on *mental health services.* There is less research on the role of *employment and welfare services* (hereby called welfare services) which are crucial for recovery in FEP through securing basic resources and employment support (Aguey-Zinsou et al., 2023; Ribanszki et al., 2022; Sylvestre et al., 2018; Wood & Alsawy, 2018). Different countries have different forms of welfare regimes. These are classified by expenditure, degree of government engagement in citizen affairs, availability of an acceptable standard of living independent of labor market performance and how the social safety net is constructed, among other classifiers (Ribanszki et al., 2022). Norway is a social democratic welfare state (Ribanszki et al., 2022) with a public health system accessible to all. This system entails both publicly funded mental health treatment and welfare assistance/support by the Norwegian Labour and Welfare Administration (NAV) (The Norwegian labour and welfare administration, 2023). NAV is a large public system that comprises both welfare and employment services which in many other countries are separate or where welfare services primarily entail means-tested social support benefits. NAV offers rights-based financial assistance such as sickness benefits and disability pension, means-tested social support benefits, as well as employment support such as work capacity assessments, internships, work-placement programs, and other employment schemes. Exploring experiences with mental health *and* welfare services in a social democratic welfare state could yield important insight into the role of services in FEP and recovery.

Finally, few studies have interviewed FEP service users in long-term as opposed to early recovery (Wood & Alsawy, 2018), although those in long-term recovery are particularly suited to report on the prolonged impact of services (Bjornestad et al., 2018; O’Keeffe et al., 2019). In our previous qualitative study on long-term recovery in FEP participants emphasized the importance of their own effort over that of services (Åsbø et al., 2024). Further exploring the complex role services have played in their recovery in the long term could have important clinical implications.

To address these gaps in our knowledge, we aimed to explore which aspects of mental health and welfare services are identified by people with lived experience of FEP as important, as well as the role of these service experiences in their long-term clinical and personal recovery.

## Method

### Context of the Study

This study is part of two catchment area-based FEP follow-up studies in Norway, the TIPS-20 (The Treatment and Intervention in Psychosis, 20-years) (Hegelstad et al., 2012) and TOP-10 (Thematically Organized Psychosis, 10-years) (Åsbø et al., 2022). Both cohorts were recruited from mental health services within the urban Oslo area. TIPS-20 participants were originally included between 1997 and 2001 within the first week of first adequate treatment. Twenty participants completed 20-year follow-up, between 2021 and 2022. TOP-10 participants were originally included in their first year of treatment between 2004 and 2012. In total, 169 participants completed 10-year follow-up, between 2015 and 2021. The current study included participants diagnosed with DSM-IV broad schizophrenia spectrum disorders or bipolar disorder with psychotic symptoms by trained psychiatrists or psychologists. The follow-up included demographic, clinical and cognitive assessment as well as extensive chart review (Åsbø et al., 2022; Hegelstad et al., 2012) (see cited studies for more information).

All participants have received treatment and support from Norway’s public health and welfare system. Treatment is defined broadly and includes, specialized and community mental health, inpatient and outpatient services. At baseline, participants in TIPS received the TIPS-project early intervention treatment (Melle et al., 2004). After first adequate treatment, most participants in TOP received outpatient treatment in specialized mental health care, which for many entailed early intervention services. Most participants have also received different types of benefits from NAV at one point during the follow-up period, such as extended sick leave and later disability pension in addition to employment support in the form of internships or work-placement programs.

### Study Design

This study is the last of a mixed methods project on long-term recovery in FEP where rates of clinical (Åsbø et al., 2022) and personal recovery (Simonsen et al., 2024) at 10-year follow-up of the TOP-study have been published. Participants from TOP-10 and TIPS-20 in clinical and/or personal recovery both by the definitions utilized in these quantitative studies (Table 1) and by their own definition where then interviewed about their long-term recovery process to learn from their experiences.

**Table 1:** Definitions of recovery

Type of recovery	Definition
Clinical recovery	Psychotic and affective symptom remission and adequate functioning for at least 12 months duration. Psychotic symptomatic remission was defined according to the RSWG (Recovery in Schizophrenia working group) (Andreasen et al., 2005) international consensus definition with scores equal to or below 3 on the following PANSS^*a*^ items at time of follow-up: positive symptoms (P1-delusions, G9-unusual thought content, P3-hallucinations), dis- organized symptoms (P2-conceptual disorganization, G5-mannerisms/posturing), and negative symptoms (N1-blunted affect, N4-social withdrawal, N6-lack of spontaneity). Discontinuation of medication is not a requirement of symptomatic remission in the consensus definition. Affective symptomatic remission was defined as an IDS-C^*b*^ score below 14, CDSS^*c*^-score below 7 and YMRS^*d*^- score below 8, as well as not meeting criteria for a current affective episode according to SCID-1^*e*^ at follow-up. Adequate functioning was defined as part-time (≥ 40%) work or study, or comparable functioning, independent living and having a close friend/confidant [18].
Personal recovery	Defined in accordance with CHIME (Leamy et al., 2011). Operationalized as a score above ≥ 45 on the 15-item version of the Questionnaire about the process of recovery (QPR) (Law et al., 2014).
Personally defined recovery (O’Keeffe et al., 2019)	Asked participants: “Do you consider yourself in recovery?” and “What does recovery mean to you?” We utilized the Norwegian word “*bedring”* in interviews, which roughly can be translated to “ongoing improvement or betterment” and describes recovery as a process. It has been previously utilized as a translation for the personal recovery process. We did not use the English word *recovery* as it is not native to the Norwegian language and may be associated with a particular perspective or recovery-oriented care in Norway.

a PANSS, Positive and Negative Symptom Scale

b IDS-C, The Inventory for Depressive Symptomatology, Clinician-rated

c CDSS, Calgary Depression Scale for Schizophrenia

d YMRS, Young Mania Rating Scale

e Structural Clinical Interview for DSM-IV Axis I disorders

These interviews were analyzed by a team consisting of clinical psychologists and researchers CS and GÅ with experience from the TOP and TIPS-study follow-ups, HH with experience from qualitative methodology and interviews regarding development and change over time, and peer-researcher SHK with lived experience and service user experience. The peer-researcher was involved in all aspects of the study and was an integrated member of the team who facilitated discussions on partial perspectives and lived experience. The analyst team met for regular research meetings over the course of a year to discuss and analyze interviews after separately reading or listening to each interview as they were transcribed (Åsbø et al., 2024).

This current phenomenological qualitative interview-study is a secondary cross-case analysis of these rich interviews regarding the long-term recovery process in FEP, where participants also reported on relevant experiences with mental health and welfare services. From the team-based analysis it was noticed that services appeared secondary to the participants’ personal resources and there were few clear tendencies in how services supported their recovery. Consequently, to better access the impact of services on long-term recovery results regarding personal resources were utilized as a conceptual framework for deductive analysis. The framework consisted of five themes from our previous paper (Åsbø et al., 2024): *Doing recovery in everyday life*; *Re-evaluating risk; Becoming a caregiver*; *Negotiating normality*; and *Owning and sharing your story*. These themes illustrate important personal developments facilitating long-term recovery in FEP related to agency, positive risk-taking, taking care of others, social context and accepting lived experience.

The epistemological framework of this study is contextualist constructivist (also called perspectivism) (Tebes, 2005), ontologically closest to critical realism. Within this perspective the participants were viewed as the experts on their experience and the truth about their recovery process as never fully accessible by researchers because this knowledge is situated and context specific and viewed through the lens of the researchers’ partial perspective (Haraway, 1988; Henwood & Pidgeon, 1994). However, by gathering several perspectives through team-based analysis as well as involving a stakeholder in the peer researcher (Henwood & Pidgeon, 1994; Tebes, 2005) the analysis aimed for a fuller picture or a “completeness of perspective” (Madill et al., 2000) of the participants’ recovery process and its facilitators.

The goal of the team-based analysis was therefore not inter-rater agreement but complementary perspectives and interpretations. Additionally, results were not seen as something that *emerged* from the material but were generated by researchers. Therein, the researcher’s perspectives were not viewed as *biases* to be removed with sufficient reflection, but something that nonetheless influenced the results. Therefore, in every analytical meeting the team members discussed reflexivity and partial perspectives especially regarding age or life stage, lived experience and experiences with mental health services. Furthermore, we frequently engaged in critical discussion of the uniqueness of the Norwegian welfare system and how this particular context might shape our interpretation and the participants’ recovery. These discussions were noted in memos from each analytical meeting to keep track of among other issues which service aspects were highlighted by each research team member.

### Participants

Among all participants from TIPS-20 and TOP-10 that were included from 2019 and evaluated to be in clinical recovery or personal recovery based on interviews and self-report (Law et al., 2014) at the time of follow-up we purposefully sampled nine participants from TIPS-20 and 11 from TOP-10. Of those asked to participate only one declined. Participants were purposefully sampled from both TIPS and TOP cohorts because they had engaged with the same services within the same catchment area, 10 years apart, allowing for a broader exploration of long-term service impact. Additionally, they were sampled for their experiences with long-term recovery as well as their extensive service experience. To enhance transferability, we also aimed to purposefully sample for heterogeneity in age, gender, diagnostic group, recovery type, employment/disability status and ethnic background to reflect the larger cohorts, although this study’s sample was somewhat less ethnically diverse.

Information power was used as the criteria for concluding sampling (Malterud et al., 2016). Due to the broad study aim, the goal to recruit for diversity and an approximate even number of participants from TOP-10 and TIPS-20 as well as the cross-case analysis aimed at catching heterogeneity of experience (Braun & Clarke, 2021) it was decided that a larger sample was necessary (Malterud et al., 2016). Information power was continually re-evaluated until it was decided that sampling could be concluded at 20 participants when the aim of the study was met.

### Interviews

Participants were interviewed in Norwegian, by GÅ, CS and SHK. Due to covid-19 restrictions (and one due to geographical location), eight were interviewed digitally using a secure video platform. The interviews were between 1.5 and 2 h in length and were audio-recorded. They were transcribed and stored securely in TSD (Services for Sensitive Data), developed, and operated by the IT Department (USIT) at the University of Oslo. Select quotations were translated to English. The participants provided written informed consent to participate in a qualitative study at follow-up, and oral consent after receiving additional information before the interview.

Interviews were based on the Life Mode Interview (Haavind, 1987; Jansen, 2015), an open interview format that is especially suited to explore the recovery process in the context of daily life. Based on an interview guide (see in supplement), we asked relevant follow-up questions about recovery and facilitators including treatment in each interview. Statements about welfare services were brought up spontaneously by the participants in most, but not all, interviews.

### Analytical Process

A secondary analysis of interviews focused on service experiences informed by the deductive analytical process described by Bingham and Witkowsky (2022) was carried out systematically in several steps by GÅ.

**Table:** 

1	Began analysis by re-reading memos from analytical meetings to re-familiarize with data and team member interpretations. Engaged in critical reflection on which service aspects were most highlighted by each researcher and their related clinical and/or service user experience.
2	Utilized NVivo 12 Pro, qualitative data analysis software to organize the data material (statements about treatment and welfare system). Created codes relevant to the research question using the conceptual framework to guide the analysis.
3	Condensed codes into topical categories of most salient and recurring aspects of service and service-user experience in line with the research aim.
4	Condensed topical categories further into five service aspects. Re-examined results at the level of codes, topical categories, and services to ensure that they appeared to adequately capture service-user experiences. Applied the conceptual framework to the data to further explain results.
5	Presented framework and preliminary results to the research-team for review, refinement and naming and renaming. Implemented suggestions and final naming of service aspects.

## Results

The 20 participants in this study have experiences with a range of services within Norway’s public health and welfare system, from specialized inpatient treatment to community care (Table 2). There was considerable variation in their engagement with services over the course of recovery. Over half of the participants had not been in contact with either mental health or welfare services for many years. Nine participants were still engaged with mental health services in the form of outpatient psychotherapy, while 14 participants, the majority, continued to be prescribed medication. Seven participants received full-time disability pension and three age-based pension from the welfare system, while the rest were employed full or part-time.

**Table 2:** Participant characteristics

	Participants (*n* = 20)
Demographic	
Gender, female	11
Age (Mdn, range)	46 (28—73)
Norwegian origin	19
In relationship	9
Have children	11
Full-time work/study	8
Part-time work/study and disability pension	2
Retired, aged based pension	3
Full-time disability pension (welfare service)	7
Clinical	
Diagnosis, 10 years	
Schizophrenia spectrum	14
Bipolar spectrum	6
Current use of medication*	14
In current outpatient treatment (mental health service)	9

*anti-psychotic/mood-stabilizer

**Fig. 1:**
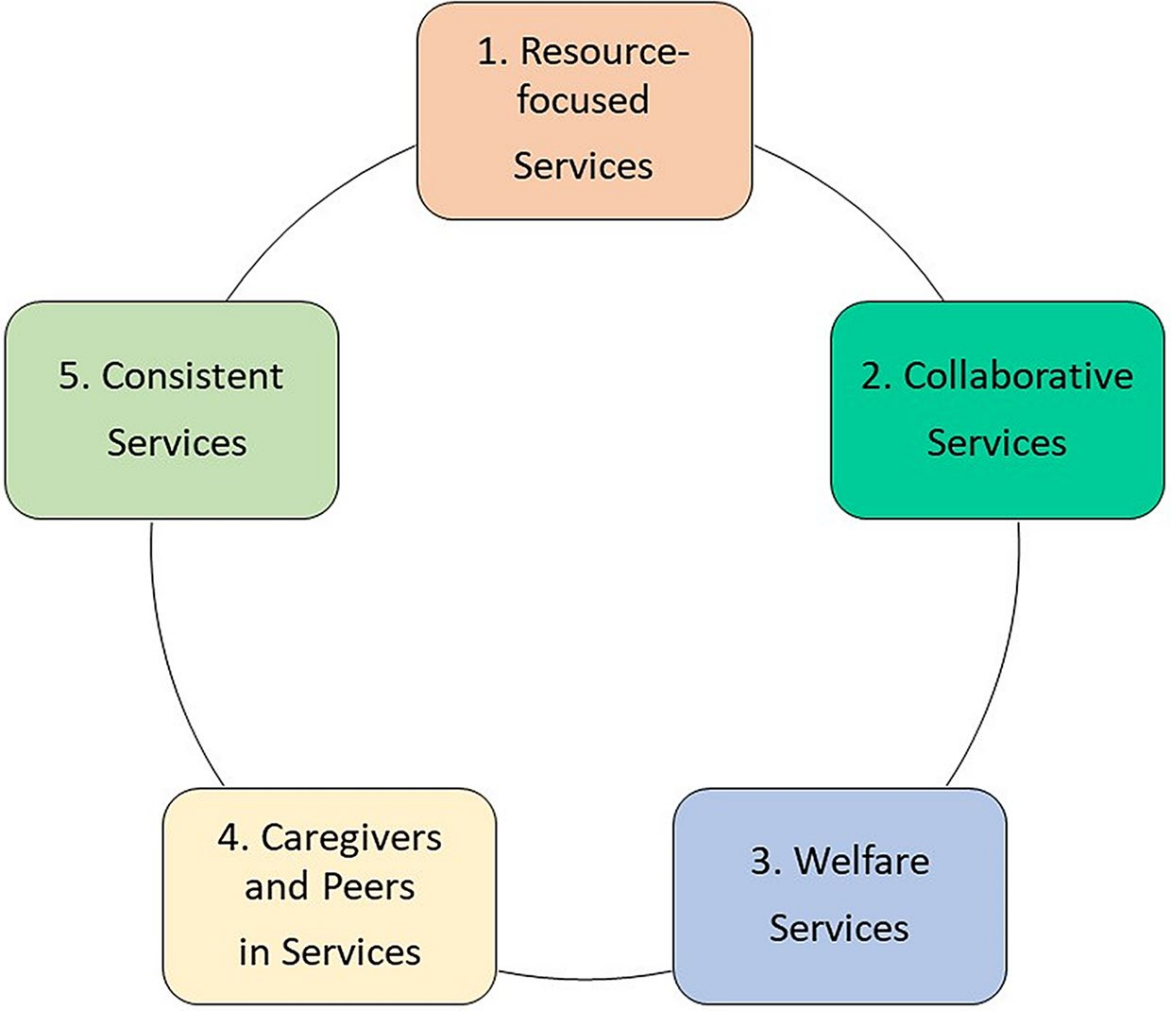
Five aspects of services important for long-term recovery in FEP

From all their accounts it appeared that the participants’ long-term recovery process as they define it began after their first adequate treatment of psychosis, as few reported on their early experiences with acute services which were thoroughly assessed in the larger follow-up studies. Furthermore, several displayed a reflective relationship to which interventions they had merely tolerated and which they had actively utilized in their recovery. However, how services were evaluated was often contradictory across, and at times within, each interview. Most participants reported some aspects of services as helpful and others as directly unhelpful or neutral, but few were described as particularly instrumental for long-term recovery. Instead, their service experiences appeared to be integrated into their larger recovery narratives where they themselves remained the main contributors to their recovery. Outside of early stabilization, services therefore seemed to play a more indirect role in recovery of supporting or hindering their development, agency and personal resources. There were no discernable differences in how participants in personal and clinical recovery evaluated services, and some, but few differences between participants diagnosed with schizophrenia or bipolar spectrum disorders in service experiences. From the deductive analysis we generated five aspects of services (see Fig. 1) impacting long-term recovery, with both positive and negative valence: *Resource-focused services*,* Collaborative services*,* Welfare services*,* Caregivers and peers in services*,* and Consistent services*. These are supported by relevant quotes (additional quotes supporting results are presented in Table 3).

**Table 3:** Quotations supporting service aspects

Service aspects	Quotes
Resource-focused services	
	“I met a doctor who empowered me a little. You don’t have a choice, but you have a choice, she said. You’re not choosing to be catatonic, but you also have a choice. When I got out after 8 weeks in the hospital I decided to get up every morning…I’m going to get better, I don’t want to deal with this anymore.” (Female, 50s)
	“I went to a new psychologist and this time we have focused on what has gone well, the positives, big or small. That did something to my thoughts. I think that has been the best treatment.” (Female, 40s)
	“I’ve gotten to know a lot of people. And I feel that they trust me. They gave me tasks that are appropriately challenging and a lot of positive feedback. And that builds self-esteem.” (Male 40s, about a community clubhouse)
Collaborative services	
Mental health services	“I want to become medication free in the long run. (…) But they have said that I need to be on medication for the rest of my life, and then I’m like, but what if I want kids or… you can’t use medications then, it’s not really tested properly” (Female, 30s)
	“I wanted a plan! (laughs) she (therapist) was more concerned with telling me that it would take time. I wasn’t interested in taking time when I got sick, I was interested in working! But no, that was like the last thing I was supposed to think about, but for me it was the most important thing. If they had helped me getting back to work I think that would have helped me get better, much quicker” (Female, 40s)
Welfare and employment services	“I was never surprised when I didn’t get a job through NAV, because my CV had more holes than a mesh shirt (…) But there was an annoying optimism at NAV, just like: just try it, it will work out! (…) It was a waste of time to put it mildly.” (Male, 20s)
Welfare services	
Financial assistance	«I was on sick leave for five years, that’s big. And I’m really happy that I got that part-time disability, I don’t know how it would have ended if I had to work full time again.” (Female, 70s)
	“I got that disability pension as a security, my finances are good and I make do.” (Male, 30s)
Disability and exclusion	“It’s easier to get to know people if you have a job and can answer all those normal things in small talk about your work. (…) I get so insecure, it would much easier if I with pride could just give a short answer. (…) I’ve thought that the day I retire I can just say: I’m retired. That would be easier. (Male, 40s)
	“Imagine if you’re totally out of commission and become one of those people that just need a lot of financial assistance from NAV, to be stuck in that system for year after year.” (Female, 40s)
Caregivers and peers in services	
Caregiver inclusion	“He (husband) wasn’t a big part of treatment, but we brought him in a few times.(…)He felt safer when he could come and talk. The provider also said that once the kids were older they could come and ask about the diagnosis and what it meant.” (Female, 50s)
	“I think my parents went there (treatment) at some point, but there wasn’t a lot of that. And they didn’t get to know anything either.” (Male, 20s)
The importance of peers	“And then we met others who had been sick too. And I got that feeling…like when you read the paper, like, oh f**k, other people have had it way worse than me. Oh s**t, that was a real wake-up call!” (Female 40s, about group therapy)
	“It has meant a lot. We have each other. (…) And we have that common experience together, what it’s like to be hospitalized. And the stigma that goes along with it.” (Female, 50s)
Consistent services	
Psychotherapy and long-term process	“What *really* made me better was the clinic with X (name of therapist). I felt relieved when I left every week. But I went for a long time. Almost five years. She was my therapist the whole time, so I have a lot to thank her for. And I think I was lucky that I got to keep her the whole time.” (Female, 70s)
	“It’s been a process. To accept that you have a mental illness is very hard. That alone took a few years. (…)
	“There was a huge turnover, I think I had nine providers in the six years I was there.” (Male, 20s)
Access to treatment after discharge	Participant: “It’s really not easy to get help. The last time I “knocked on the door” of the clinic they said, you have to go back down to the ER. Gosh, I was really sick, so I was afraid of going to the ER. (…) So it’s a little illogical getting help here in Norway, it’s kinda hard.”
	Interviewer: “What do you wish it was like?”
	Participant: “More accessible (…) Or that they have a list…like if you’re on hold in a call queue, that they have to prioritize the people that are really struggling.” (Male, 40s)
	«I need a SHRINK! (laughs). (…) You’re supposed to go through your GP, but where can you get help? I mean, what can you do? (…) I got my third rejection from a psychologist because the wait list was too long, there were 150 people on the waitlist.” (Male, 30s)

### Resource-Focused Services: Focused on Personal Strengths Rather than Limitations

*Resource-focused services* illustrates that interventions which supported their strengths seemed to result in a sense of hope and agency that was the starting point of recovery for several participants. Some reported feeling empowered by providers who expected something of them and others from specific interventions. Volunteer or community services that offered practical interventions, such as activity planning, not explicitly tied to symptoms or illness were reported as helpful by some participants:

“start to work with everything that’s… just ordinary (…) build up self-esteem, build up all the things that makes the psychosis take up less space. (…) I have a contact person in the community that I talk to weekly. We don’t talk much about illness, but about structure, routine, establishing an ordinary life that will make me capable of being in a relationship.” (Male, 30s).

Another participant was devastated when she could not return to work after her first episode and felt “useless”. She experienced a sense of accomplishment and forward movement during a knitting class in occupational therapy that motivated her to continue her process:

“at the hospital they had occupational therapy, so I could come there to knit and sew every day. It sounds so corny, but it was really good for me! (…) occupational therapy helped me feel mastery, and it had nothing to do with what someone said, because it was something I experienced on my own.” (Female, 30s).

*Resource-focused services* provided a wide range of interventions that focused on strengths instead of deficits, created hope and empowered some participants to continue to work on long-term recovery in their everyday life.

### Collaborative Services: Based in Shared Decision-Making and Transparency

*Collaborative services* illustrates that the service experience most participants felt had hindered their recovery was a lack of collaboration or shared decision-making. Contrary to the empowerment some experienced discussed above, several participants seemed to have been met with low expectations and little opportunity to influence their treatment. This issue appeared in several types of services and interventions but was mainly discussed within outpatient treatment as few reported on inpatient treatment.

When asked about experiences with treatment, participants mainly reported on outpatient psychosocial treatment and fewer on experiences with medication. Many participants did not experience their psychosocial treatment as significant for recovery because there was little transparency and collaboration around its purpose. From participant accounts collaboration also depended on a competent helping professional providing adequate information and expertise, as providers were often described as “nice” but not particularly helpful. The most common complaint was that the treatment did not adequately address the participants’ subjective recovery goals, typically about working or studying. One participant was thankful for her multi-service treatment team, but called them a “pain in the ass” because they made her doubt herself:

“It’s been a pain in the ass on one hand and a nice thing on the other. It feels like I’m treated to death in a way. (…) even though they say that I’m good enough I’m also hearing you can’t do this, you shouldn’t do that.” (Female, 30s).

The power differences between service user and provider also presented itself in a lack of transparency or psychoeducation about diagnosis and chances for recovery:

Participant: “I didn’t even hear the word psychosis from a doctor. So what I want to bring up today…a piece of advice, there’s a bit of…not degradation, but unfortunate treatment of people in a situation like that (serious mental illness). There should be more educative treatment, that you get proper information.”

Interviewer: “Psychoeducation?”

Participant: “Yes, psychoeducation! I didn’t even know that word.” (Female, 60s).

Participants appeared to have received better information and more structured follow-up regarding outpatient medication than in their psychosocial treatment:

“They don’t ask: what’s important to you?! (…) I didn’t get a chance to tell them how I was doing, it was more like, how are the meds? And that was good! I felt heard when they agreed that it was good for me to taper carefully. (…) I experienced good collaboration around medication.” (Female, 50s).

Medications were largely described as helpful for recovery because they provided a sense of safety and stability, while some participants described negative experiences and large side-effects from compulsory medications. In long-term recovery discontinuing anti-psychotic or mood-stabilizer medication appeared to be a goal or concern for several participants. Some worried about long-term consequences and others seemed to view discontinuation as a final proof of recovery. However, it differed how much collaboration they experienced around tapering and discontinuation from providers.

Difficulties with collaboration were also discussed within the Norwegian Labour and Welfare Administration (NAV), as two participants joked about the well-known inflexibility of their rights-based benefit systems:

“When NAV says: jump, I say: how high? (laughs)” (Male, 40s).

Several participants also reported that NAV work internships were irrelevant to their interests and skill-level, and therefore did not lead to sustained employment. Some also wished for better collaboration or coordination between services regarding employment.

In *Collaborative services* a lack of shared decision-making seemed to have made some participants disengage from treatment and employment support or feel hindered in pursuing long-term recovery goals.

### Welfare Services: Offering Safety through Basic Resources and Employment

*Welfare services* illustrates that help from both mental health and welfare services with accessing basic resources have been instrumental for many participants’ recovery, as welfare can mean both government benefits and wellbeing. Several participants reported that receiving financial assistance from welfare services was crucial because they could live good lives and focus on recovery without the added stressors of housing and food insecurity. Sickness benefits allowed some to return to work after longer absences and made for a more inclusive labor market, while long-term disability supported stability for others:

“Without worrying that I don’t’ have a job or enough money…That’s more important than not having symptoms.” (Female, 50s).

However, the participant discussing financial security above continued her reflection by stating that *not* working felt even more excluding than being diagnosed with a psychotic disorder:

“not being able to work has been worse than the symptoms. I had difficulty handling why I wasn’t working, because it was something I felt that I should be able to do. (…) That was hard to tell people, that’s been one of the hardest things.” (Female, 50s).

Employment was reported by several participants as one of the most important facilitators of their recovery because it provided financial security, a sense of purpose, and social inclusion. Receiving disability pension was therefore connected to considerable stigma, exclusion, or a sense of not contributing to society for several participants:

“For me, being recovered is to work (…) If I wouldn’t be able to work, I would be nothing, a burden to society (…) It made me feel even sicker, in a way, and useless…” (Female, 40s).

*Welfare services* have contributed to long-term recovery because receiving financial assistance or employment support have helped many participants live good lives. However, for some participants, permanent disability was experienced as a double-edged sword which both provided security but also acted as a barrier to fully participate in society.

### Caregivers and Peers in Services: Including the Experts

*Caregivers and peers in services* illustrates that the participants’ social support network had been instrumental for their recovery, but they were rarely included in treatment. The participants’ caregivers had frequently encouraged them to seek help, advocated on their behalf and helped them get back on their feet at the start of recovery. However, this significant resource was under-utilized by services as few had received family therapy. One participant stated that her family, and especially father who was her greatest support, did not receive adequate information from the treatment team when she was hospitalized:

“It was my dad that came to visit every day at the hospital when I was first committed (…) That was hard for him too. The first time I was hospitalized he was told that I would never recover (laughs in disbelief)” (Female, 40s).

In addition to their family, several participants reported that they received support from peers with lived experience who provided fellowship, understanding, and functioned as role-models the participants could learn from. Some participants considered using their own lived experience as a resource to help others. The value of peers also became apparent in some of the interviews, as one participant said that it was much easier to share his lived experience with the peer-researcher who interviewed him than providers. Nevertheless, no participant had met a formal peer-support worker:

“The friends I made in the hospital were more of a support than psychologists and psychiatrists. (…) to use people with the same experiences in treatment, I think that is a really smart and important step in the right direction.” (Female, 40s).

Including *Caregivers and peers in services* therefore appear important for long-term recovery because their support and encouragement of help-seeking and further growth would have been a significant resource for services.

### Consistent Services: Providing Long Term Follow up with a Consistent Aim

*Consistent services* illustrates that several participants benefited from long-term treatment for long-term recovery, but this was often hindered by structural barriers and a lack of continuity in care. This was brought up by many participants when they reflected on what would have been helpful to them in their current stage of recovery after discharge from early intervention services. Several conveyed that they were still in need of some follow-up but were *too recovered* to qualify for services in the public system and wished for a more streamlined route back into care:

“I’m falling between two chairs. I’m too recovered to treat, but too sick to deal with life.” (Male 30s).

Long-term, consistent psychotherapy was one of the few specific types of treatment several participants highlighted as helpful for recovery. However, some mentioned structural barriers to the consistency they needed such that the treatment was too infrequent, or the therapist turnover was too high. Additionally, that the treatment lacked a consistent aim or purpose. Some expressed that they needed time to process challenging experiences of psychosis and mania and how they had affected their sense of self:

“But if it’s really serious, like the things you’re researching (psychotic disorders), then you often need longer therapy. That you get to work through the matter over many years and don’t get released after three months to end up as a revolving door patient for the rest of your life.” (Male, 30s).

*Consistent services* appear important for long-term recovery because many participants expressed that they were still in need of some support or follow up after discharge. Long-term consistent therapy was especially valuable because it provided many participants with an avenue for sharing, processing, and accepting their lived experience.

## Discussion

This qualitative study’s 20 participants in long-term recovery in FEP are all previous or current service users. From their accounts, being met with respect, collaboration and continuity in care seemed to have a larger influence on their recovery long-term than specific interventions. These results are consistent with other qualitative studies on mental health care and FEP (Hansen et al., 2020; O’Keeffe et al., 2018; Skjærpe et al., 2023; Wood & Alsawy, 2018) and recovery-oriented practice (Boutillier, 2011; Jaiswal et al., 2020; Leamy et al., 2011).

It has been suggested that one barrier to the consistent implementation of recovery-oriented practice (Jaiswal et al., 2020; Wood & Alsawy, 2018) is that service users are not seen as “*equal partners in the evaluation of treatment”* (Van Eck et al., 2018, p. 639). The participants in this study were skilled evaluators who could reflect on which aspects of treatment they had just taken or accepted, and which they had made active use of. They also appeared to view treatment as less instrumental for their long-term recovery than their own efforts, where the role of services was more indirect through mobilization of their personal resources.

This result is not inconsistent with the importance of long-term care or early intervention in FEP (Bighelli et al., 2021; Leendertse et al., 2021; Temesgen et al., 2019; Wood & Alsawy, 2018) as we have interviewed people *in* recovery who have received extensive treatment and assistance. Rather, the participants demonstrate that consistent support can result in a narrative development from service user to a person who has taken charge of recovery in their own life. The five service aspects discussed below were reported as experiences in the participants overall recovery narratives and therefore add nuance to previous research that explores whether types of treatment help or hinder recovery (Temesgen et al., 2019; Wood & Alsawy, 2018). However, to our knowledge no previous study has explored long-term recovery and FEP service user experiences with welfare services, where all service aspects are also relevant.

Resource-focused services offered hope as well as practical interventions that empowered some participants to begin their recovery process, often in community or low-threshold services. The importance of hope, empowerment and holistic services is found in previous studies (Boutillier, 2011; Jaiswal et al., 2020; Temesgen et al., 2019; Wood & Alsawy, 2018). Our results suggest that welfare services through providing employment support (Borg et al., 2013; Khoronzhevych et al., 2022) as well as a resource-oriented attitude towards service users (Irvine & Haggar, 2023) can also contribute to empowerment, where the latter is less frequently researched.

Many participants reported on their experiences of being met with low expectations and paternalism in services, a common experience for people with psychotic disorders (Britz & Jones, 2023; Thornicroft et al., 2022). This was reported equally by participants in personal and clinical recovery, although a previous FEP study found that those in clinical recovery had more positive treatment experiences (O’Keeffe et al., 2022). Improving collaboration and shared-decision making is therefore a key target for services to aid clinical and personal recovery in FEP and a strikingly consistent finding in service user research (Fusar-Poli et al., 2022; O’Keeffe et al., 2018; Slade, 2017).

Few participants brought up experiences of coercive inpatient treatment compared to other studies (Fusar-Poli et al., 2022), although most participants in this study have experienced involuntary comittal. Furthermore, other prioritized aspects of recovery-oriented services such as human rights-based and trauma-informed care were not reported on by participants (World Health Organisation, 2021). However, the participants in this study are largely white of Norwegian national origin, reside in a well-built public health and welfare system and are in recovery by their own definition and by criteria, which all might indicate more favourable service experiences (Hui et al., 2021; Jones et al., 2024). More research on recovery and involuntary treatment is necessary alongside reviews on personal recovery in different social contexts (Douglas et al., 2022; Slade et al., 2012).

The Norwegian Labour and Welfare Administration (NAV) played a crucial role in the participants’ recovery because benefits allowed them to live good lives regardless of labor market participation. According to a recent review (Ribanszki et al., 2022) social democratic welfare systems are superior for most mental health outcomes compared to other welfare models. Specific to SMI is that far fewer experience absolute poverty and homelessness after FEP in these systems (Ljungqvist et al., 2016; Sylvestre et al., 2018). Yet, there is a dearth of research on recovery and service user experiences with welfare services.

Although this social democratic welfare model with its social safety net is crucial for ensuring financial security and access to treatment for people with complex needs, people with psychotic disorders continue to have among the highest disability rates and rates of NEET (not in education, employment, or training) status, also in Norway (Aguey-Zinsou et al., 2023; Ajnakina et al., 2021; Evensen et al., 2016). The low employment rate in FEP is partially related to psychosocial functioning (Gühne et al., 2022) but previous quantitative studies from our project suggest that it is not solely explained by illness related impairment as the majority are in long-term remission (Åsbø et al., 2022; Tandberg et al., 2013). Several systemic barriers to employment in FEP are also implicated including discrimination and poor accommodations (Harkko et al., 2023; OECD, 2013; Thornicroft et al., 2022).

Norway has been highlighted by the OECD as having particular barriers to employment of people with SMI (OECD, 2013). These include the welfare system in itself (OECD, 2013) through the phenomenon occasionally labelled as “the welfare trap” (Ilmakunnas, 2023). This mechanism is complex and includes duration dependence where long-term social assistance recipiency reduces chance of exiting assistance (Ilmakunnas, 2023). Additionally, having few prospects outside low-paid short-term work after FEP might reduce the incentive to pursue employment instead of a secure income through benefits. However, one study from Germany found that the majority of unemployed FEP-participants desired to work (Gühne et al., 2021).

A larger issue attributable to the welfare system is perhaps that some are prematurely placed on permanent disability after FEP before receiving adequate employment support (Aguey-Zinsou et al., 2023; Jones et al., 2023). A few participants still struggled to envision that they could work or study even after 10 years in stability. A qualitative study found that barriers to employment in early psychosis is a complex process of derailment of careers, lowered expectations from caregivers and services leading to lowered expectations of oneself and missing vocational identity (Jones et al., 2023). Furthermore, this and other studies have discussed the self-labelling of a “disability mindset” or “disablement process” (Blajeski, 2020; Jones et al., 2023) that can occur for people with psychotic disorders when receiving disability pension.

As such, the high disability rate in FEP is detrimental to recovery because as participants reported not working is attached to stigma and further social exclusion (Aguey-Zinsou et al., 2023; Blajeski et al., 2021; OECD, 2013). Researching and addressing systemic barriers to employment in FEP is therefore crucial (Marwaha et al., 2013). Furthermore, participants expressed receiving little support with employment after FEP from both mental health and welfare services meaning that both services are important to address the high rate of disability in FEP (Aguey-Zinsou et al., 2023; Harkko et al., 2023; Jones et al., 2023). Improved coordination between mental health and welfare services (OECD, 2013) in promoting access to employment and education through for instance individual placement and support (IPS) (Aguey-Zinsou et al., 2023; Hegelstad et al., 2019) therefore appears instrumental for recovery and equal citizenship (Boutillier, 2011).

Results demonstrate that service users’ social support network should be better included in treatment, even in a strong public health system such as Norway that is less reliant on informal caregivers (Abou Seif et al., 2022). Caregivers and peers have been called invisible experts and experts by experience, respectively, due to their unique insights about the service user and what they might need (Abou Seif et al., 2022; McLaughlin, 2009). Their support and knowledge are therefore important resources for mental health services. Family therapy is one of the most effective psychosocial interventions in psychosis (Bighelli et al., 2021), although it was not as routinely offered in standard outpatient psychosis services in Norway a decade ago when some of the participants received treatment (Langeveld et al., 2024). The importance of peer-support work is also well known, but continues to be under-utilized (Bighelli et al., 2021; Jaiswal et al., 2020). Family and peers are important for encouraging employment after FEP (Blajeski, 2020; Blajeski et al., 2021) and therefore a likely resource for employment and welfare services as well.

Long-term treatment, and especially therapy was the psychosocial intervention that most participants reported as helpful for long-term recovery. Recent studies have suggested a need for more in-depth psychotherapy for psychotic disorders to process ineffable experiences and potential associated trauma or grief (Fusar-Poli et al., 2022; Ridenour et al., 2023). A finding we did not explore further was that participants with bipolar disorder seemed more dissatisfied with psychosocial treatment. Early intervention services have been less adjusted to accommodate people with bipolar disorders (Conus & McGorry, 2002) for whom treatment is often primarily focused on stabilization and psychoeducation (Kahn et al., 2004). Similarly, continuity in care was frequently hindered by structural barriers such as premature discharge or difficulty accessing treatment, which have been reported by FEP service users to hinder recovery (Boydell et al., 2010). As such, consistent implementation of recovery-oriented early intervention and community services should be a priority, and could be more cost-effective long-term according to the World Health Organization (WHO) (World Health Organisation, 2021).

### Service Implications

The main service implication of this study is that there is no singular intervention that will promote recovery in FEP for all, but what is offered requires continuity in care. Shared decision-making is essential to find what will be helpful to each individual service user at a given time and is as dependent on service user feedback as a competent, but transparent provider. The clear information and consistent structure offered in outpatient medication follow-up also appear helpful for psychosocial treatment. Furthermore, medication discontinuation appears to be a common concern in long-term psychosis/affective stability that should be addressed in treatment. In line with personalized medicine, holistic and tailored interventions that focus on service users’ resources seem beneficial. These should also include family therapy and peer-support. The implications are largely in line with recovery-oriented care and are relevant for both mental health and welfare services. Therein, better service coordination regarding employment support and financial stability appears crucial for recovery.

### Strengths and Limitations

This study provides important knowledge on the role of services in the long-term recovery of participants within two rigorous FEP follow-up studies, with some methodological strengths and limitations. A main strength is that including a broad FEP spectrum as well as a peer-researcher enhances transferability and potential resonance of results (Carminati, 2018; Levitt et al., 2018). In addition, the more comprehensive open interview format informed by the Life Mode interview allowed the participants to report more freely on service experiences as a part of their rich recovery narratives (Davidson, 2003), a substantial strength of the study. We have not systematically compared the interviews conducted by the three interviewers or further explored any implications of using multiple interviewers. The interviewers had varying experience, different conversational styles, ways to build rapport, and perhaps pursued different topics which likely influenced results. Nonetheless, there were few obvious differences in the participants’ accounts that were easily attributable to the interviewer. This could be due to utilizing the Life Mode interview where *every* interview is different and largely led by the participant as opposed to a semi-structured interview where questions are the same across. One potential implication and strength of using multiple interviewers is that participants perhaps felt safer to share some experiences or critical reflections regarding services with the peer-researcher, which one participant confirmed in the interview. However, due to the more open interview format and analysis grounded in personal recovery experiences we can comment less on the role of specific interventions. A deductive analysis could also narrow results compared to more data-driven, inductive approaches. The sample was also ethnically homogenous and few reported severely negative service experiences, which could leave out other valuable service user accounts (Hui et al., 2021). Future research should include qualitative studies that explore the impact of various types of treatment as well as experiences with welfare services with methods beyond-semi structured interviews. Many of our results are consistent with the literature but not sufficiently implemented in services. Therefore, crucial areas for future research are implementation science and influencing policy makers.

### Conclusion

To conclude, from this analysis of FEP service-user experiences the overarching result is that for people in long-term recovery the long-term impact of services lies in how their further development beyond stability is supported. Furthermore, that in both mental health and welfare services, relational aspects of collaboration, consistent support, and inclusion of the people around them were important for recovery. Collaboration also extended to a need for better coordination between mental health and welfare services. We hope these results will encourage future research, and most importantly, more consistent service implementation.

## Electronic supplementary material

Below is the link to the electronic supplementary material.

Supplementary Material 1

## Declarations

### Pre-registration

This study was pre-registered in the Open Science Framework: https://osf.io/pmqv6/.
