# Supplementary material for: Taking what you get or Getting what you Need: A Qualitative Study on Experiences with Mental Health and Welfare Services in Long-Term Recovery in First-Episode Psychosis
Source: Community Ment Health J. 2024 Oct 16;61(2):350–64. doi: 10.1007/s10597-024-01356-6 (PMC11772452; doi:10.1007/s10597-024-01356-6)
Supplement: Supplementary file 1 — Supplementary Material 1 [file 10597_2024_1356_MOESM1_ESM.docx]

# Supplementary material

# Taking what you get or getting what you need: a qualitative study on experiences with mental health and welfare services in long-term recovery in first-episode psychosis

Gina Åsbø*^1,2,3^, Hanne Haavind^3^, Sindre Hembre Kruse^2^, Kristin Fjelnseth Wold^1,4^, Wenche ten Velden Hegelstad^5,6^, Kristin Lie Romm^2,1^, Mike Slade^7,8^, Torill Ueland^1,3^, Ingrid Melle^1,4^, Carmen Simonsen^2,1,3^

^1^ Section for Clinical Psychosis Research, Department of Research and Innovation, Division of Mental Health and Addiction, Oslo University Hospital, Oslo, Norway

^2^ Early Intervention in Psychosis Advisory Unit for Southeast Norway, Division of Mental Health and Addiction, Oslo University Hospital, Oslo, Norway

^3^ Department of Psychology, Faculty of Social Sciences, University of Oslo, Oslo, Norway

^4^ Institute of Clinical Medicine, University of Oslo, Oslo, Norway

^5^ TIPS – Centre for Clinical Research in Psychosis, Stavanger University Hospital, Stavanger, Norway

^6^ Faculty of Social Sciences, University of Stavanger, Norway

^7^ School of Health Sciences, Institute of Mental Health, University of Nottingham, Nottingham, UK

^8^ Faculty of Nursing and Health Sciences, Health and Community Participation Division, Nord University, Namsos, Norway

*Corresponding author:

email: [gina.asbo@psykologi.uio.no](mailto:gina.asbo@psykologi.uio.no)

ORCID: 0000-0003-3135-3522

## Interview Guide

- **Interview method**:
  - Life Mode interview (Haavind, 1987; Jansen, 2015):
    - Example of questions asked in a typical Life Mode interview: “can you walk me through your daily routine, for instance, where did you wake up yesterday? and what did you do after you woke up?” Adding questions about living situation, social circle, activities, typical routine and uncommon events
    - Connecting questions on everyday life to development and process, for instance: “how long have you..?”, “has it always been like this?” “this seemed like an important event to you?” “what has changed?” “what made this possible?

Specific questions related to recovery will be integrated into the Life Mode interview if needed. Participants will be asked to deliberate when they bring up relevant topics to research aim, or if no such topics are discussed by the participants, questions below can be utilized. Original interview guide was created in Norwegian and then translated to English for this supplement.

|  | **Interview questions**  Main question:   - Potential follow-up question, if necessary |
| --- | --- |
| **How is the recovery process described?** | From your own understanding of what recovery is, do you consider yourself recovered?”   - How do you know? - When did you know?   How would you describe your recovery process?   - Has anything been particularly challenging? - Have things turned out differently than you imagined? - What have you learned from the recovery process? - How happy are you with where you are now? Can anything improve?   How do you picture yourself and your life in the future? |
| **What has been important for recovery?** | What has been important for your recovery?  How have you yourself contributed to your recovery?   - What do you think about this now? Would you have done anything differently? - Has this changed how you see yourself, and how?   How have the people around you contributed to your recovery?   - What do you think about this now? Could they have done anything differently?   How have you experienced the mental health system and how has it contributed to your recovery?   - Has it hindered your recovery in some way? - Has any type of treatment been more helpful than others? Which? - How could the treatment further aid recovery? - Did you receive enough treatment? |
| **How is recovery defined?** | What does recovery mean to you? |
|  | Is there anything else you would like to add? |

**References**

Haavind, H. (1987). *Liten og stor: mødres omsorg og barns utviklingsmuligheter [The big and the little one: Maternal care and the developmental possibilities for children]*. Universitetsforlaget.

Jansen, A. (2015). Positioning and subjectivation in research interviews: why bother talking to a researcher? *International Journal of Social Research Methodology*, *18*(1), 27–39. https://doi.org/10.1080/13645579.2013.845711
